# Assessing awareness in severe Alzheimer’s disease

**DOI:** 10.3389/fnhum.2022.1035195

**Published:** 2023-02-01

**Authors:** Jonathan Huntley, Daniel Bor, Feng Deng, Marco Mancuso, Pedro A. M. Mediano, Lorina Naci, Adrian M. Owen, Lorenzo Rocchi, Avital Sternin, Robert Howard

**Affiliations:** ^1^Division of Psychiatry, University College London, London, United Kingdom; ^2^Department of Psychology, University of Cambridge, Cambridge, United Kingdom; ^3^School of Psychology, Trinity College Dublin, Global Brain Health Institute, Dublin, Ireland; ^4^Human Neuroscience Department, Sapienza University of Rome, Rome, Italy; ^5^Department of Physiology and Pharmacology, Brain and Mind Institute, University of Western Ontario, London, ON, Canada; ^6^Department of Psychology, Brain and Mind Institute, University of Western Ontario, London, ON, Canada; ^7^Institute of Neurology, University College London, London, United Kingdom

**Keywords:** awareness, consciousness, EEG, fMRI, TMS

## Abstract

There is an urgent need to understand the nature of awareness in people with severe Alzheimer’s disease (AD) to ensure effective person-centered care. Objective biomarkers of awareness validated in other clinical groups (e.g., anesthesia, minimally conscious states) offer an opportunity to investigate awareness in people with severe AD. In this article we demonstrate the feasibility of using Transcranial magnetic stimulation (TMS) combined with EEG, event related potentials (ERPs) and fMRI to assess awareness in severe AD. TMS-EEG was performed in six healthy older controls and three people with severe AD. The perturbational complexity index (PCI^ST^) was calculated as a measure of capacity for conscious awareness. People with severe AD demonstrated a PCI^ST^ around or below the threshold for consciousness, suggesting reduced capacity for consciousness. ERPs were recorded during a visual perception paradigm. In response to viewing faces, two patients with severe AD provisionally demonstrated similar visual awareness negativity to healthy controls. Using a validated fMRI movie-viewing task, independent component analysis in two healthy controls and one patient with severe AD revealed activation in auditory, visual and fronto-parietal networks. Activation patterns in fronto-parietal networks did not significantly correlate between the patient and controls, suggesting potential differences in conscious awareness and engagement with the movie. Although methodological issues remain, these results demonstrate the feasibility of using objective measures of awareness in severe AD. We raise a number of challenges and research questions that should be addressed using these biomarkers of awareness in future studies to improve understanding and care for people with severe AD.

## 1. Introduction

There are currently 50 million people living with dementia globally, of whom an estimated 12%, are in the severe stages of the disease (Prince et al., 2014). A person who lives for 10 years with AD spends an average of 40% of this time with severe AD (Arrighi et al., 2010). People with severe AD have extensive cognitive deficits, impairing language and communication and there is often a lack of recognition of relatives and apparent unawareness of time, place or features of their environment. Although there is a growing literature on person-centered care (Kim and Park, 2017; Fazio et al., 2018) people living with severe AD remain a vulnerable, under-researched and neglected group (Profyri et al., 2022). A challenge for healthcare services and family members is the uncertainty of how best to care for people with advanced dementia and how to improve their wellbeing and quality of life. This uncertainty is directly related to a lack of understanding of the lived experience of people with advanced dementia. What are they able to experience of the world around them? What are they aware of? Do delivered interventions and care make any difference to them? In the absence of any reliable biomarkers, clinicians and caregivers often make assumptions about the level of awareness of a person with severe dementia, based on observations of behavior. This has resulted in conflicting reports of awareness in people with severe dementia (Clare, 2010), and uncertainty as to the extent of self-awareness, or awareness of the environment at the end stages of AD (Clare, 2010; O’shaughnessy et al., 2020). There is a perception of the need to enrich environments in care homes to stimulate and engage patients (Prince et al., 2013), but no clear evidence for how this should be done in an evidence-based manner to improve the experience and outcomes of people with severe dementia. Of crucial importance for the wellbeing of people with dementia and for health and care services is whether the potential expression of awareness may be dependent on, or modified by, external factors including more need-sensitive care (Clare, 2010; Clare et al., 2013). There is, therefore, an urgent need to understand and measure awareness in advanced dementia, and how this may be impacted by interventions and care.

Consciousness is a multifaceted concept that includes two major components: *wakefulness* (i.e., the level of consciousness) and *awareness* (i.e., the contents of consciousness) (Laureys, 2005; Boly et al., 2013). Observational clinical tools are available to assess the level of consciousness (Giacino et al., 2004), and the study of awareness in healthy people, and in people with mild to moderate AD, has largely relied upon patients verbally reporting their subjective experiences (Cosentino and Stern, 2005). These subjective reports remain the “gold-standard” approach to assessing awareness, however, are difficult or impossible in people with moderate to severe dementia, who may no longer be able to communicate reliably. Recent advances in consciousness science have introduced a number of new methods and biomarkers for assessing awareness in people who cannot communicate their subjective experience (Owen et al., 2006; Cruse et al., 2011; Casali et al., 2013; Naci et al., 2014). This article describes these approaches and their application to assessing awareness in severe AD.

A combination of transcranial magnetic stimulation (TMS) and electroencephalography (EEG) has recently been used to assess the capacity for consciousness in non-communicative patients in vegetative and minimally conscious states (Napolitani et al., 2014). TMS is a non-invasive technique that stimulates the cerebral cortex using a brief magnetic pulse applied to the scalp. This induces focal neuronal discharge at the cortex surface and EEG can then measure cortical electrical responses, both locally and at distant sites. In this way, using TMS pulses the brain can be briefly perturbed, and the resulting spatiotemporal activity patterns can be analyzed to measure the dynamical complexity of brain activity, referred to as the “perturbational complexity index” (PCI) (Casali et al., 2013). PCI is intended to capture the simultaneous occurrence of two properties of brain activity: integration (all regions of the brain respond cohesively as a whole), and differentiation (responses are diverse and heterogeneous). The co-existence of integration and differentiation has long been conjectured to be the basis of consciousness (Tononi et al., 2016), which aligns well with PCI’s experimental results: conscious subjects display widespread temporally complex responses to TMS, resulting in high PCI values, while in unconscious subjects, brain responses remain local to the stimulation site or are temporally stereotypical, resulting in low PCI values (Casali et al., 2013).

Empirically, PCI has been demonstrated to reliably characterize and discriminate between conscious states in different clinical conditions (e.g., vegetative states and anesthesia) and during different stages of sleep (Casali et al., 2013). Multiple variants of PCI have been put forward (Casali et al., 2013; Comolatti et al., 2019), and we use the PCI^ST^ method by Comolatti et al. (2019), which is fast, simple, and can be applied to low-density EEG (Comolatti et al., 2019). This makes it ideal for clinical applications, including the potential use outside a research facility, such as in a care home. The benefit of this approach for assessing consciousness in people with advanced AD, is that it does not rely on the integrity of sensory or motor pathways, does not require any response or action from the participant, and can differentiate between different conscious states at an individual level. To our knowledge, our feasibility and pilot work reported here is the first application of PCI to people with severe AD.

In order to isolate the neural correlates of the content of consciousness, experimental paradigms have traditionally relied on our ability to report our conscious experiences. Neurophysiological or functional neuroimaging methods are used to contrast brain activity associated with stimuli that are reported to be consciously experienced from those that are not. For example, visual stimuli can be experimentally manipulated to be consciously seen or not seen using a range of methods including masking, flash suppression, binocular rivalry or change blindness (Dehaene and Changeux, 2011). One commonly used method involves electroencephalography (EEG) to measure event related potentials (ERPs) while participants view target stimuli (e.g., faces) presented between a series of non-target masks. The conscious perception of faces can be manipulated depending on the duration of presentation, with awareness of “seeing a face” emerging when the face is presented for approximately 100 ms (Del Cul et al., 2009). According to one predominant model of consciousness, the global neuronal workspace theory (GNWT), visual stimuli that are consciously experienced and reported are associated with “ignition” and activation of a fronto-parietal network, which amplifies and sustains a neural representation of the stimuli (Mashour et al., 2020). GNWT predicts that these neural mechanisms provide a global workspace for information to be maintained and accessed by a range of brain networks, to enable conscious processing. Measuring ERPs in occipital and parietal electrodes in response to consciously perceived visual stimuli reveals an initial positive component at approximately 100 ms, a negative component at around 200 ms known as the visual awareness negativity (VAN), and a positive component at around 300 ms known as the late positivity (LP) (Rutiku et al., 2016). There remains controversy as to which of these ERP components are necessary and/or sufficient for consciousness, and which may be associated with preconscious processing or the reporting or manipulation of conscious content (Lamme, 2018). Nonetheless, the robust data on ERP components that are commonly associated with conscious experience of visual stimuli act as an important starting point for assessing conscious experience in clinical populations that are unable to communicate (Harrison and Connolly, 2013).

Similarly, neuroimaging studies in humans and primates have demonstrated that functional connectivity between a distributed system, including primary sensory, parietal and frontal cortices, plays a crucial role in the generation of perceptual awareness (Laureys, 2005; Dehaene et al., 2006; Rees, 2007; Koch et al., 2016). A network of prefrontal and parietal regions is also associated with higher-level awareness of self and judgments of performance in healthy adults and in AD (Fleming and Dolan, 2012; Lou et al., 2017; Hallam et al., 2020).

Functional magnetic resonance imaging (fMRI) can also demonstrate covert awareness in patients who are unable to provide a verbal or behavioral response (Owen et al., 2006; Owen and Coleman, 2008). A recent study reported that watching a short engaging film leads to patterns of brain activity across auditory, visual and fronto-parietal networks that are synchronized across individuals (Naci et al., 2014). The time series within the frontoparietal network has been shown to reflect the individual’s conscious experience of the film, varying with how engaging the plot is from scene to scene as well as with subjective measures of suspense. The common pattern of activation within the frontoparietal network across healthy individuals, has been considered to be a “biomarker” for the conscious experience associated with watching the film. This biomarker can then be used to assess the presence of conscious experience in individual behaviorally unresponsive patients, by measuring its similarity over time to that of healthy, conscious individuals. This approach has demonstrated preserved frontoparietal activation in response to a film in an individual who was assumed to be in a vegetative state, but who was subsequently shown to have intact perceptual and higher-level awareness (Naci et al., 2014).

These methods therefore provide objective biomarkers of awareness that could provide evidence for the capacity for consciousness, as well as a window into the subjective experience of severe AD. This article sets out to:

1) Examine the feasibility of using TMS-EEG, ERP and fMRI to assess awareness in people with severe AD.
2) Describe preliminary data on TMS-EEG based metrics of the capacity for consciousness in severe AD.
3) Describe preliminary data on whether there is ERP and fMRI evidence of brain activity that reflects preservation of higher level awareness in people with severe AD.

## 2. Materials and methods

We conducted three case-control feasibility and pilot studies comparing TMS-EEG, ERP, and fMRI markers of awareness in people with severe AD and healthy controls. Participants with severe AD, classified by the clinical dementia rating scale (CDR) (Morris, 1997) and Global deterioration scale (Reisberg et al., 1982) and healthy non-dementia participants were recruited. As participants with severe AD lacked capacity to consent, following the legal framework of the Mental Capacity Act (2005), personal consultees were identified and provided a declaration that the person would have wished to take part in the study (HRA, 2017). The study was approved by the Wales three NHS ethics committee. Methods are outlined below and further details of participants, data acquisition and analyses are provided in the online supplementary material (see Supplementary material).

### 2.1. Assessing capacity for consciousness using TMS-EEG

Single-pulse, monophasic TMS (>150 trials) was delivered using a Magstim 2002 device connected to a 70-mm figure-of-eight coil targeted to the vertex. Each participant’s resting motor threshold (RMT) was calculated, and the stimulation intensity was set at 120% RMT. EEG was recorded from 63 active electrodes mounted on a cap (actiCAP). Activity was averaged across all artifact-free trials to obtain a TMS-evoked potential between 0 and 300 ms post-TMS, and PCI^ST^ was computed using publicly available open-source code,^1^ so that the results are directly comparable to those reported by Comolatti et al. (2019).

### 2.2. Assessing visual perception using event related potentials

**FIGURE 1:**
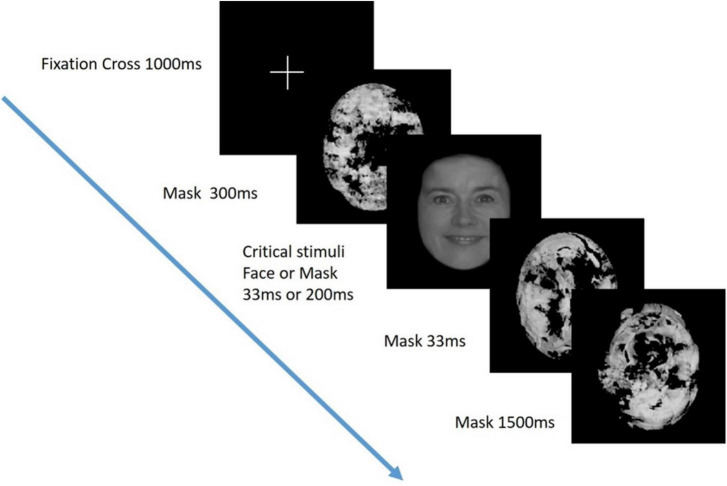
Visual perception task. Critical stimuli (either a face or mask), were presented at either 33 ms or 200 ms duration in a visual masking paradigm adapted from Kouider et al. (2013). Previous studies have demonstrated that stimuli presented at 200 ms but not 33 ms are consciously perceived.

A visual masking paradigm was adapted from a study of visual awareness in infants, who similar to people with severe AD, are also unable to provide verbal reports of their conscious experience (Kouider et al., 2013; Figure 1). A series of black and white pictures, either of faces (the “target”) or masks, were presented at either 33 ms or 200 ms duration. The targets or masks were presented in 10 pseudo-random blocks of 20 trials (5 trials of 4 trial types). Participants were positioned 30 cm from a computer screen on which the stimuli were presented and monitored to ensure their eyes were open and gaze was focused on the screen during the task. ERPs were recorded using the same 63 active electrode EEG system as in the TMS experiment.

### 2.3. Assessing awareness using fMRI

We presented an edited version of the black and white TV episode, “Alfred Hitchcock Presents - Bang! You’re Dead” as used in previous studies by Naci et al. (2014). Imaging data was collected using a Siemens 3T scanner, functional echo-planar images were acquired during the movie, and an MPRAGE anatomical volume was obtained. Participants were monitored using an infrared camera inside the scanner to ensure spontaneous eye opening during the movie. Data-driven analysis of fMRI time series was conducted using established auditory, visual and frontoparietal components associated with healthy brain function during movie-viewing (Naci et al., 2014, 2017), To identify these components in study participants we performed group ICA (Calhoun et al., 2001), a method that derives spatially orthogonal components, whose spatial and temporal features are similar across subjects. Previous studies using this method have focused on the robustness of three main networks in auditory, visual and frontoparietal regions (Naci et al., 2014). Each network’s time course (derived from the ICA of the healthy group) was then used as a regressor in the SPM data model of the AD patient. If the patient’s brain activity in frontal and parietal regions is tightly correlated with the healthy participants over time, such functional correspondence can be interpreted as demonstrating conscious awareness during movie-viewing at a single-subject level (Naci et al., 2014).

## 3. Results

A summary of demographic information is shown in Table 1. The healthy control participants were younger than the people with advanced dementia (73.4 vs. 80.5 years). The participants with dementia were all classified as in the severe stage of AD using the Clinical dementia rating scale (sum of boxes) (mean 17.5, SD 1) and Global deterioration scale (6.8 SD 0.5). Control participants had no cognitive decline (CDR 0; GDR 1). Two severe AD patients have both PCI and ERP data and one patient was unable to tolerate the length of the session and therefore only TMS-EEG data was collected. None of these three participants were able to tolerate fMRI. The participant who underwent fMRI was a different participant who was unable to have TMS-EEG due to a history of seizures.

**TABLE 1:** Demographic information.

	Controls (*n* = 8)	Severe AD (*n* = 4)
Age (mean, SD)	73.4 (4.7)	80.5 (2.4)
Gender F/M	4/4	3/1
CDR-SB (mean, SD)	0 (0)	17.5 (1)
GDS (mean, SD)	1 (0)	6.8 (0.5)

CDR-SOB, clinical dementia rating scale—sum of boxes—scored out of 18 (16–18 = severe); GDS, global deterioration scale—scored out of 7 (6–7 = severe).

### 3.1. TMS-EEG

**FIGURE 2:**
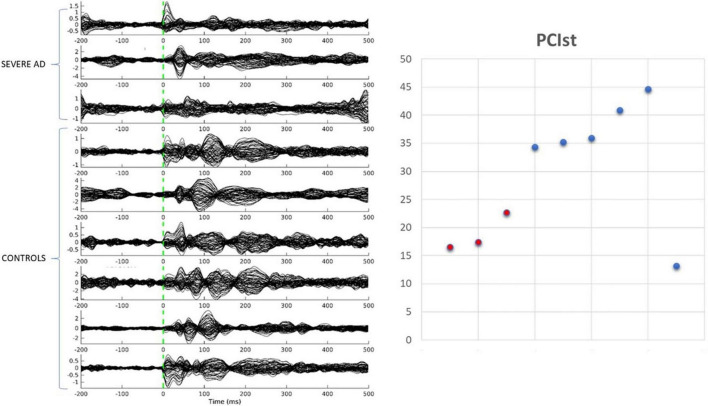
Transcranial magnetic stimulation evoked potentials and PCIst values for control participants and patients with severe AD.

We collected data from seven healthy older adults and three people with severe AD. One of the healthy participants had very few trials remaining after cleaning and was excluded from the analysis. As shown in Figure 2, five of the six healthy controls had a PCI^ST^ ranging between 34.4 and 44.6, in line with the healthy adults described by Comolatti et al. (2019). One healthy participant had an unusual result (for a healthy participant) of 13.2. The three patients with severe AD had PCI^ST^ values of 16.5–22.7. In the Comolatti et al. (2019), study the threshold for consciousness was measured to be around 21.25 for 19 channel EEG (Comolatti et al., 2019), suggesting that patients with AD are around or slightly below the threshold for consciousness. However, further data is required to fully document the normal variability of PCI^ST^ scores for both healthy older adults and people with severe dementia.

### 3.2. ERP

**FIGURE 3:**
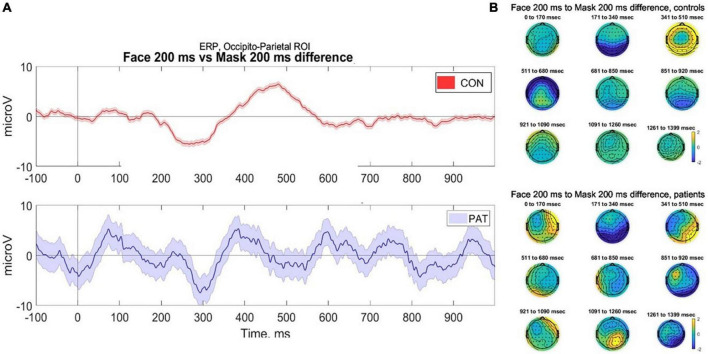
**(A)** Averaged voltage across six occipito-parietal electrodes. Controls (top) demonstrate classical components associated with facial perception, namely the visual awareness negativity (VAN) at 200 ms and late positivity (P400) during stimuli where faces were presented at 200 ms, compared to control (masked) trials. In contrast, participants with severe AD (bottom) demonstrate evidence of VAN but reduced late positivity, however due to small numbers the error bars are wide for the patients. **(B)** 2D scalp topographies of the EEG activity in response to facial perception (trials of face presented for 200 ms). EEG signal is averaged on grand average based time-windows. CONS, controls; PAT, people with severe dementia.

In line with Kouider et al. (2013) we examined eight occipito-parietal electrodes (O2-Oz-O1-POz-PO3-PO4-PO8-PO7) to identify ERP components associated with face perception. We averaged the voltage across the considered cluster of electrodes and plotted amplitude vs. time (sec) (Figure 3).

Data were collected from seven healthy older participants and two participants with severe AD. The mean number of artifact-free trials was 49.5. Due to the small number of participants, statistical analysis of the components was not conducted; therefore any conclusions are preliminary and require validation. As shown in Figure 3, in response to faces presented at 200 ms, healthy controls demonstrated a robust VAN and LP response, compared to masked trials and those at 33 ms duration. This is in keeping with the literature that conscious perception is associated with VAN and LP components (Rutiku et al., 2016). In contrast, the participants with severe AD provisionally demonstrated a VAN, but a potentially reduced LP response to the face presented at 200 ms. This pilot data demonstrates the feasibility of collecting ERP data using a passive visual masking paradigm with participants with severe dementia; however, due to the very small sample size the error bars for the AD patients are wide and more data is required before conclusions can be made.

### 3.3. FMRI

**FIGURE 4:**
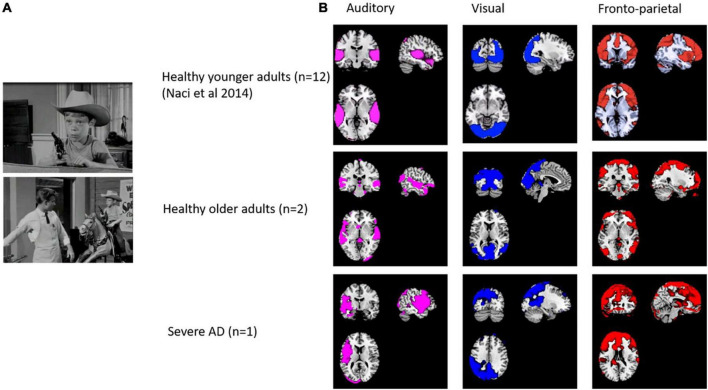
**(A)** Stills from the edited movie “Bang! You’re dead!”. **(B)** Spatial comparison of auditory, visual, auditory and fronto-parietal components in 12 healthy adults (from Naci et al., 2014), older adults (*n* = 2) and a patient with severe dementia.

We collected pilot data from two healthy older participants and one participant with severe AD. As shown in Figure 4, the ICA revealed strong components in auditory, visual and frontoparietal networks in older participants and in the participant with severe AD. Some differences in localization are expected due to morphological differences (e.g., widespread atrophy and enlarged ventricles) in the patient compared with controls, as well as the normal anatomical variation observed even among individual healthy participants. However, despite ICA demonstrating spatially similar components, time series analysis demonstrated no significant correlations for the components between patients and older or younger controls. This demonstrates the feasibility of collecting fMRI data in people with severe AD and that activity beyond auditory and visual networks including fronto-parietal activity, can occur in severe AD. However, the lack of temporal synchronization of brain activity between the severe AD patient and healthy participants requires further research. More age- matched control and patient data is required to assess whether the lack of correlation of fronto-parietal activity suggests differences in conscious experience of the movie in severe AD.

## 4. Discussion

These three experiments demonstrate that using these methods in people with severe AD is feasible. The results demonstrate the potential for assessing the capacity for consciousness, and the contents of perceptual consciousness in people with severe AD. Although the PCI and fMRI paradigms have been validated at the single subject level in other clinical groups, the small number of participants in the study mean that these preliminary findings need to be interpreted with caution and need to be validated in future studies. The identification of biomarkers for level and content of consciousness raise multiple research questions for future studies (see Box 1).

**BOX 1 Future research questions and validation experiments.**

1) What is the range of consciousness biomarkers seen in people with severe AD, as measured using markers of dynamical complexity e.g., PCI?
2) Does capacity for consciousness (PCI values) fluctuate within individuals with severe AD?
3) Does capacity for consciousness (PCI values) differ according to internal factors (e.g., level of arousal) and/or external factors (e.g., type or salience of stimuli).
4) Do apparent moments of lucidity reported in people with severe AD (e.g., in response to music) represent genuine fluctuations in consciousness, measurable using biomarkers?
5) Do different subtypes of AD (e.g., PCA) or other common forms of dementia (e.g., bvFTD, DLB) differ in biomarkers of awareness and how is this associated with phenomenological differences and underlying neuropathology?
6) How does impairment in content of consciousness relate to decline in executive function, attention and episodic memory in early AD?
7) How robust are these preliminary findings of a reduced LP and preserved VAN in severe dementia? Does a reduced LP suggest that people with severe dementia have preserved phenomenal consciousness but impaired access consciousness?
8) Do PCI, ERP and fMRI markers differ between mild, moderate and severe AD, and do any differences relate phenomenologically to changes in subjective awareness?
9) Can behavioral interventions (e.g., cognitive stimulation, music therapy, and exercise) enhance arousal and awareness in moderate and severe AD?
10) Do common BPSD (e.g., agitation, apathy, and psychotic symptoms) relate to variability in arousal and awareness that can be measured using EEG and fMRI markers?
11) Could pharmacological approaches to enhance arousal and attention affect awareness in people with dementia?

Although this study demonstrates the feasibility of assessing awareness using TMS-EEG, ERP, and fMRI in severe AD, considerable challenges remain. A significant challenge is the interpretation of biomarkers in the absence of a gold standard measure of awareness, due to the unreliability of verbal or behavioral responses in people with advanced dementia. Without this benchmark, it may remain unclear whether biomarkers are accurate, however, as in the growing literature on the use of biomarkers in disorders of consciousness (DoC), these markers of awareness in dementia patients can be compared to the performance of healthy participants and other clinical groups under similar experimental conditions (Peterson et al., 2015). Several studies have demonstrated statistically significant findings using TMS-EEG, ERP and fMRI markers of awareness in the majority of participants. For example, an empirical PCI cutoff has discriminated with 100% accuracy between conscious and unconscious conditions, irrespectively of connectedness, responsiveness and presence of brain lesions (Casarotto et al., 2016). These methods have been used to differentiate and explore consciousness in a range of clinical conditions (Harrison and Connolly, 2013; Arsiwalla and Verschure, 2018; Sinitsyn et al., 2020; Sarasso et al., 2021) and have laid a conceptual groundwork to enable the extension of these markers to people with advanced dementia. It is possible, however, that there are additional confounds when applying these markers, validated in DoC, to people with dementia, due to the cognitive impairment and neurodegeneration that characterizes dementia. It remains unclear how changes in cognitive domains, such as executive function, attention and episodic memory may correlate or relate causally to phenomenological changes in awareness, however this does not invalidate the use of these biomarkers as a measure of awareness. We remain agnostic as to the contribution and overlap between awareness and cognitive processes, however it is likely that there is a correlation between impaired awareness and cognitive decline in AD (Huntley et al., 2021). Future work in people with mild and moderate dementia, including longitudinal multimodal assessments may help clarify these issues. Similarly, the ongoing work to identify neural correlates of conscious (NCCs) specifically aims to identify the brain processes (structural, functional, and electrophysiological) that are necessary and sufficient for consciousness (Crick and Koch, 1998). The nature or extent of neurodegeneration in NCCs that may result in changes in awareness in dementia remains unclear, however it is likely that AD pathology impacting neural networks identified as associated with awareness results in phenomenological deficits in awareness in AD (Hallam et al., 2020; Huntley et al., 2021). The use of brain-based markers of awareness in people with DoC with varying degrees of brain damage and heterogenous pathologies sets a precedent for using these measures of awareness in dementia, despite not having a definitive answer to the precise NCCs. Nonetheless, methodological issues remain, for example there is evidence that false TMS-EEG readings can result from TMS delivered at the site of localized brain lesions (Gosseries et al., 2015). Similarly, cortical atrophy in advanced dementia presents methodological challenges in ensuring the TMS pulse produces a significant TEP to enable meaningful analysis. Improving the quality and reproducibility of data using real time monitoring of TEPs (Casarotto et al., 2022) may reduce confounders from artifact and poor data quality and enable PCI markers to be more clinically reliable, however challenges remain as demonstrated in our study by participants who are unable to tolerate neuroimaging to support accurate neuronavigation of the TMS pulses. Associated with these issues is the inadequate characterization and heterogeneity of people with severe AD. When people lose the ability to communicate clearly or cooperate with assessment, as was the case for participants in our study, we have been reliant on observational descriptions of their behavior and function. Whilst some validated qualitative tools exist to assess awareness [e.g., “AwareCare” (Clare et al., 2013)] there is a need for more clinically useful, multidimensional quantitative behavioral measures of awareness to complement neurophysiological biomarkers. Such tools should quantify verbal, behavioral, physiological and social indicators of awareness. This would enable a triangulation of approaches to characterize the multidimensional nature of consciousness.

One unexpected result was the demonstration of a low PCIst value in a healthy, conscious individual. It is possible that this may relate to an inadequate TEP, or may represent that despite monitoring, the participant entered a transient microsleep state as a result of fluctuating levels of vigilance, as is frequently observed during resting-state experiments (Tagliazucchi and Laufs, 2014; Demertzi et al., 2019). As discussed above, the interpretation of the phenomenology accompanying PCI or other markers in unresponsive people remains even more speculative, as it is not possible to collect data on experience during data acquisition (Demertzi et al., 2019). For example, as PCI values for individuals during REM sleep may also be within the range seen in awake individuals (Casali et al., 2013), it is possible the finding of a preserved PCI value in severe AD may not represent capacity for conscious awareness connected to the environment, but rather a hallucinatory experience disconnected from the external world.

Whilst these pilot data demonstrate the feasibility of investigating residual awareness in severe AD, challenges therefore remain to identify the quality and content of such awareness, and interpretation of the data relies on the predictive power of current theoretical models and the validity of neural correlates of consciousness. The methods are drawn from major theories of consciousness, namely the integrated information theory (IIT) (Tononi et al., 2016) and global neuronal workspace theory (GNWT) (Dehaene et al., 2011). These models have been successful in providing experimental methods for identifying neural correlates of consciousness (NCCs). In turn, the identification of NCCs enables inferences to be made regarding the level and content of consciousness in clinical groups who are unable to communicate their conscious experience. However, controversies remain. For example, within visual awareness, there is debate as to whether the VAN or LP are both true NCCs, or whether the VAN is associated with phenomenal consciousness, whilst the LP reflects access consciousness or is related to the processing of conscious information by working memory or attentional processes (Lamme, 2018). In contrast it may be that the VAN does not represent consciousness at all, but denotes pre-conscious processing, as the GNWT may predict? There remains ongoing debate and research into which ERP components represent true NCCs, and which may instead represent pre, or post conscious processing (Aru et al., 2012). Questions remain regarding the richness of the subjective experience in severe dementia, for example whether the ERP and fMRI markers suggest phenomenal or lower level awareness, and to what extent access to higher level consciousness may persist or fluctuate. It will be important in future studies to examine the variability of these ERP markers seen in advanced dementia, and also in mild-moderate dementia. By combining ERP markers with information on the extent and distribution of structural and functional changes due to neurodegeneration in AD a picture of how AD affects these brain-based markers can be developed.

As future studies build a clearer picture of the range of electrophysiological and neuroimaging markers of awareness in AD, this will enable an increased understanding of how consciousness is affected as AD progresses. It will also reciprocally enable AD to act as a complex lesion study for consciousness and allow the investigation of the interaction between markers of consciousness, neurodegeneration and decline in related higher-level cognitive domains. Importantly the conceptualization of consciousness and awareness in AD must recognize the centrality of psychosocial theories of personhood. The neuroscientific investigation of consciousness must link to the wider social, interpersonal, cultural and spiritual dimensions underpinning awareness and personhood, which cannot be reduced to biomarkers. The crucial questions of what people with dementia are aware of and how they experience themselves and the world around them cannot be easily answered with current technology. The valuable information regarding consciousness gained from neuroscientific methods, can be used to compliment the holistic view of the person with dementia and harnessed to improve understanding, care and preserve the humanity and dignity of people with severe dementia.

## 5. Conclusion

A clearer understanding of consciousness in severe AD is urgently required to improve care. By demonstrating feasibility and reporting objective markers of awareness in severe AD for the first time we have set out future research and validation experiments to further understand the central issue of conscious experience in AD.

## Data availability statement

The raw data supporting the conclusions of this article will be made available by the authors, without undue reservation.

## Ethics statement

The studies involving human participants were reviewed and approved by the Wales 3 NHS Ethics Committee. The patients/participants provided their written informed consent to participate in this study. As participants with severe AD lacked capacity to consent, following the legal framework of the Mental Capacity Act (2005), personal consultees were identified and provided a declaration that the person would have wished to take part in the study (HRA, 2017).

## Author contributions

JH, DB, LN, AO, LR, and RH contributed to conception and design of the study. JH, DB, PM, FD, MM, and AS performed the statistical analyses. JH wrote the first draft of the manuscript. DB, AS, PM, and LR wrote sections of the manuscript. All authors contributed to manuscript revision, read, and approved the submitted version.

## Footnotes

1
https://www.github.com/renzocom/pcist
