# Supplementary material for: Assessing awareness in severe Alzheimer’s disease
Source: Front Hum Neurosci. 2023 Feb 1;16:1035195. doi: 10.3389/fnhum.2022.1035195 (PMC9930987; doi:10.3389/fnhum.2022.1035195)
Supplement: Supplementary file 1 [file Data_Sheet_1.pdf]

Supplementary Material

1 Supplementary Methods

1.1 Participants and recruitment

Participants with severe AD, and healthy non-demented older control participants were recruited from memory services in Camden and Islington NHS Foundation Trust and using the national NIHR Join Dementia Research platform. For participants with AD, pre-existing diagnoses were previously made by NHS services according to ICD-10 diagnostic criteria (WHO, 2016), following multidisciplinary assessment. Patients were eligible for inclusion if they had a diagnosis of F00.1 (Dementia in Alzheimer disease with late onset) or F00.2 (Dementia in Alzheimer disease, atypical or mixed type), as pathologically, mixed AD neurodegeneration and cerebrovascular disease remain common in people with clinically diagnosed AD (Arvanitakis et al., 2019). As a definitive diagnosis of Alzheimer's disease remains a post-mortem diagnosis, the participants were diagnosed as 'probable' AD following standard clinical assessment, and severity of dementia was defined using established cut-off scores on the standardised mini mental state examination (sMMSE): severe (< 10) (Folstein et al., 1975), Clinical dementia rating scale (Morris, 1997) and Global Deterioration Scale (Reisberg et al., 1982) Exclusion criteria included any concurrent medical condition, psychiatric illness or medication that may interfere with awareness and a history of epilepsy or metallic implants would contraindicate the person's ability to undergo the TMS-EEG or fMRI, component of the study. As participants with severe AD lacked capacity to consent, strict procedures for recruitment and consent of patients unable to provide informed consent were followed (HRA, 2017), and personal consultees were identified and provided a declaration that the person would wish to take part in the study. The study was approved by the Wales 3 NHS ethics committee. Of note, the same older

control participants were used in both the TMS-EEG and ERP experiments. Two patients with severe AD had both TMS-EEG and ERP data collected, whilst one patient only had TMS-EEG data collected. Two different healthy older controls and one different severe AD patient took part in the fMRI experiment. Please see Table 1 for demographic information of all participants. In addition, the three severe AD patients who had TMS-EEG data had been diagnosed with probable AD 10 years, 8 years and 4 years prior to the study. The severe AD patient who took part in the fMRI study was diagnosed with probable AD 10 years prior to the study, with repeat neuroimaging 1 year prior to the study suggesting additional cerebrovascular disease leading to a revised diagnosis of F00.2: Dementia in Alzheimer disease, atypical or mixed type.

1.2 Experiment 1 TMS-EEG

1.2.1 Data Acquisition: Participants were sitting in a comfortable chair, in a quiet room, resting their forearms on a pillow placed on their lap. They were asked to fixate on a point on the computer screen, in order to reduce eye movements during the EEG recordings. Participants wore headphones, which continuously played a masking noise, composed of white noise mixed with specific time varying frequencies of the TMS click, to minimize auditory evoked potentials. To enhance the masking, subjects also wore ear defenders (SNR = 30) on top of the earplugs. The intensity of the masking noise was adjusted for each participant by increasing the volume (always below 151 dB) until the participant was sure that s/he could no longer hear the TMS click (Rocchi et al., 2021). Single-pulse, monophasic TMS was delivered using a Magstim 2002 device connected to a 70-mm figure-of-eight coil held with the handle backwards at 45 degrees to the midline, inducing current in the posterior-anterior direction (Magstim Company Limited, Whitland, UK). Resting motor threshold (RMT) was calculated as the lowest stimulation intensity that produced a motor evoked potential of at least 50 μV in 5 out of 10 consecutive trials in the relaxed first dorsal interosseous

muscle. When using the standard coil, the stimulation intensity was set at 120% RMT. EEG was recorded using a DC-coupled, TMS-compatible amplifier (Actichamp, Brain Products, GmbH). Signal was recorded from 63 active electrodes mounted on a cap (actiCAP), in accordance with the international 10-10 system, including: Fp1, Fz, F3, F7, FT9, FC5, FC1, C3, T7, TP9, CP5, CP1, Pz, P3, P7, O1, FCz, O2, P4, P8, TP10, CP6, CP2, Cz, C4, T8, FT10, FC6, FC2, F4, F8, Fp2, AF7, AF3, AFz, F1, F5, FT7, FC3, C1, C5, TP7, CP3, P1, P5, PO7, PO3, POz, PO4, PO8, P6, P2, CPz, CP4, TP8, C6, C2, FC4, FT8, F6, AF8, AF4, F2. Recordings were online referenced to Oz and the ground electrode was placed on Fpz. In the offline analysis, an average reference was used. Skin impedances were kept below 5 kΩ and the sampling frequency during recording was 5000 Hz. Additionally, when the coil was in direct contact with the EEG cap, a 0.5 cm foam layer was placed underneath the coil to minimise bone conduction of the TMS click and scalp sensation caused by coil vibration.

1.2.2 Data analysis, Preprocessing: Offline EEG pre-processing was performed with EEGLAB 14.1.1 with additional functions included in the TMS-EEG Signal Analyser (TESA) toolbox (Rogasch et al., 2017), using MATLAB (version 2020a). EEG signal was epoched (-1.3 to 1.3s) and demeaned using a baseline from -1000 to -10ms. Channels that exceeded a kurtosis threshold were considered faulty and excluded from further analysis. Next, inspired by the Autoreject method, peak-to-peak amplitudes for each channel and epoch were computed (excluding 20ms around the TMS pulse), and epochs with either multiple channels exceeding a soft threshold, or at least one channel exceeding a hard threshold, were rejected. Finally, all channels were linearly detrended using the slope estimated from the 50 to 500ms range post-TMS.

With bad channels and epochs removed, the remaining artefacts were cleaned using an adapted version of the SOUND-SSPSIR pipeline available in the TESA toolbox, which we describe here in brief. First, the TMS pulse was removed from -5 to 2ms around the trigger and interpolated by means

of a cubic function. Independent component analysis was then run using the FastICA algorithm to remove blink and movement artefacts, and the remaining artefacts were removed using the SOUND (Mutanen et al., 2018) and SSP-SIR (Biabani et al., 2019) algorithms. Lastly, data was band-pass filtered between 0.1 and 45Hz with a fourth-order Butterworth filter, resampled at 362.5Hz and rereferenced to the common average reference.

1.2.3 PCI calculation: We computed PCI^{ST} using publicly available open-source code by Comolatti et al (2019), which we describe here in brief (Comolatti et al., 2019). First, data from all artefact-free trials is averaged to obtain the TMS-evoked potential (TEP) in all channels. Then, the principal components (PCs) of the TEP are obtained, and for each PC a pairwise distance matrix is obtained for the baseline (-200 to –50ms) and response (0 to 300ms) time ranges. These matrices are thresholded to obtain the number of state transitions in the signal, and the optimal threshold is selected as that which maximises the difference in state transitions between response and baseline. Finally, the process is repeated for each PC and the resulting number of state transitions are summed across PCs to yield the final PCI^{ST}. For more details on the computation of PCI^{ST} please see the original publication (Comolatti et al., 2019).

1.3 Experiment 2: Event related potentials

1.3.1 Data acquisition: A visual masking paradigm was adapted from a study of visual awareness in infants (Kouider et al., 2013). A series of black and white pictures, either faces or masks, were presented at either 33ms or 200ms duration. The target face or mask were presented between a series of masks in 10 pseudo-random blocks of 20 trials (5 trials of 4 trial types). Participants were positioned 30cm from a computer screen on which the stimuli were presented and monitored to

ensure their eyes were open and gaze was focused on the screen during the task. ERPs were recorded using the same 63 active electrode EEG system as in the TMS experiment.

1.3.2 Data analysis: Offline EEG pre-processing was performed with EEGLAB 14.1.1, running using MATLAB (version 2018b). EEG signal was epoched (-1.7 to 1.7s) using a baseline from -1200 to -400ms, in order to avoid considering the EEG response to the fixation mask as baseline. A band-pass (1-100Hz) and band stop (48-52Hz) filter was applied and the signal was further epoched (-1 to 1s) to reduce possible edge artefacts. Epochs were then visually inspected and those with excessively noisy EEG were excluded. Independent component decomposition analysis was run, using a fastICA algorithm, and components were plotted in a time window ranging from -500 to 1000ms. Those representing artefacts (eyeblinks, continuous muscle activity etc) were removed. Lastly, EEG signals were rereferenced to the common average reference. In line with Kouider et al 2013 we examined eight occipito-parietal electrodes (O2-Oz-O1-POz-PO3-PO4-PO8-PO7) to identify ERP components associated with face perception. We averaged the voltage across the considered cluster of electrodes and plotted amplitude vs time (sec). Due to the small number of participants in this pilot study, statistical analysis of the components was not conducted. No conclusions can therefore be drawn however the amplitude vs time plots were produced to allow preliminary illustrative comparison between groups that requires validation with larger sample sizes in future experiments.

1.4 Experiment 3: fMRI

1.4.1 Data acquisition: The design was based on previous studies by Naci et al 2014 and 2017 (Naci et al., 2014, Naci et al., 2017). An edited 8-min sequence of the black and white TV episode, "Alfred Hitchcock Presents - Bang! You're Dead" was presented. Participants were asked to simply watch the film, which was projected on to a screen behind their head, and they looked upward into a mirror

box that enabled them to see the screen. An infrared camera placed inside the scanner was used to monitor the participants to ensure they maintained continuous eye opening during the movie. Noise cancellation headphones were used to deliver sound delivery. An anatomical volume was obtained using a T1 weighted 3D magnetization prepared rapid acquisition gradient echo (MPRAGE) sequence (32 channel coil, voxel size 1 x 1 x 1mm, TA = 5 min and 38s, TE=4.25ms, matrix size = 240 x 256 x 192, FA = 9 degrees). During the movie, functional echo-planar images were acquired [48 slices, voxel size: 3 x 3 x 3, repetition time = 3360ms, matrix size = 64 x64, flip angle (FA) = 75 degrees)

1.4.2 Data analysis: Data were processed using automatic analysis (version 4.1) (Cusack et al., 2014): a data processing and analysis pipeline that integrates commonly used routines from Statistical Parametric mapping (SPM12) with custom data processing modules written in MATLAB. Five 'dummy' scans were excluded from the beginning of every data set. Data were normalized to MNI space and smoothing was done with a Gaussian kernal of 10mm FWHM. The standard preprocessing steps were corrected for timing of slice acquisition, motion correction, normalization to a template brain, and smoothing. Motion correction was done using six motion parameters (x,y,z, translation and rotation) and low-frequency noise (e.g., drift) was removed with a high-pass filter of 128s. To further denoise the data, while maintaining the temporal integrity the data, cerebrospinal fluid, white matter signals, motion parameters, their lag-3 2nd-order volterra expansion, and "spikes" (based on mean signal variance across volumes) were used as nuisance regressors. The data were then further cleaned by running a group ICA (Calhoun et al., 2001) within each stimulus and removing 1-2 components that spatially correlated with a mask of the ventricles to remove non-brain related activity.

1.4.3 Data-driven analysis of fMRI time series: a model of healthy brain function during movie viewing has been established in previous studies (Naci et al., 2014, Naci et al., 2017), demonstrating that individual components clustered into five spatially distinct brain networks: auditory, visual, frontoparietal, motor and precuneus. To identify these components in study participants we performed group ICA, a method that derives spatially orthogonal components, whose spatial and temporal features are similar across subjects. The GIFT software (https://trendscenter.org/software/gift/) was used to perform ICA with a 15-component cutoff. The components corresponding to the networks of interest (visual, auditory, frontoparietal) were visually identified.

Similar to the approach used by Naci et al 2014, the model of healthy brain function during the movie task, based on the stereotypical brain activity observed in healthy participants, could be used to compare brain function in individual patients. Single-subject analyses were focused on three main networks, the auditory, visual and frontoparietal which are functionally critical for higher-order cognition during movie viewing. Each network's time course (derived from the ICA of the healthy group) was then used as a regressor in the SPM data model of the patient. The spatial similarities of the identified networks can be used to determine functional correspondence between the healthy controls, older adults, and AD patient. Slight differences in localization are expected not only due to differences in any given patient's morphological organisation (e.g. widespread atrophy, enlarged ventricles etc) compared with controls but also due to the normal anatomical variation observed even among individual healthy participants. By comparing the time courses of activation in response to the film from each of the identified networks, homologous processes between the healthy controls, older adults, and AD patient, can be identified.

References:

ARVANITAKIS, Z., SHAH, R. C. & BENNETT, D. A. 2019. Diagnosis and Management of Dementia: Review. *JAMA,* 322**,** 1589-1599.

BIABANI, M., FORNITO, A., MUTANEN, T. P., MORROW, J. & ROGASCH, N. C. 2019. Characterizing and minimizing the contribution of sensory inputs to TMS-evoked potentials. *Brain Stimul,* 12**,** 1537-1552.

CALHOUN, V. D., ADALI, T., PEARLSON, G. D. & PEKAR, J. J. 2001. A method for making group inferences from functional MRI data using independent component analysis. *Hum Brain Mapp,* 14**,** 140-51.

COMOLATTI, R., PIGORINI, A., CASAROTTO, S., FECCHIO, M., FARIA, G., SARASSO, S., ROSANOVA, M., GOSSERIES, O., BOLY, M., BODART, O., LEDOUX, D., BRICHANT, J. F., NOBILI, L., LAUREYS, S., TONONI, G., MASSIMINI, M. & CASALI, A. G. 2019. A fast and general method to empirically estimate the complexity of brain responses to transcranial and intracranial stimulations. *Brain Stimulation*.

CUSACK, R., VICENTE-GRABOVETSKY, A., MITCHELL, D. J., WILD, C. J., AUER, T., LINKE, A. C. & PEELLE, J. E. 2014. Automatic analysis (aa): efficient neuroimaging workflows and parallel processing using Matlab and XML. *Front Neuroinform,* 8**,** 90.

FOLSTEIN, M. F., FOLSTEIN, S. E. & MCHUGH, P. R. 1975. "Mini-mental state". A practical method for grading the cognitive state of patients for the clinician. *J Psychiatr Res,* 12**,** 189-98.

HRA. 2017. *Consent and Participant Information Guidance* [Online]. NHS Health Research Authority. [Accessed].

KOUIDER, S., STAHLHUT, C., GELSKOV, S. V., BARBOSA, L. S., DUTAT, M., DE GARDELLE, V., CHRISTOPHE, A., DEHAENE, S. & DEHAENE-LAMBERTZ, G. 2013. A neural marker of perceptual consciousness in infants. *Science,* 340**,** 376-80.

MORRIS, J. C. 1997. Clinical dementia rating: a reliable and valid diagnostic and staging measure for dementia of the Alzheimer type. *Int Psychogeriatr,* 9 Suppl 1**,** 173-6; discussion 177-8.

MUTANEN, T. P., METSOMAA, J., LILJANDER, S. & ILMONIEMI, R. J. 2018. Automatic and robust noise suppression in EEG and MEG: The SOUND algorithm. *Neuroimage,* 166**,** 135-151.

NACI, L., CUSACK, R., ANELLO, M. & OWEN, A. M. 2014. A common neural code for similar conscious experiences in different individuals. *Proc Natl Acad Sci U S A,* 111**,** 14277-82.

NACI, L., SINAI, L. & OWEN, A. M. 2017. Detecting and interpreting conscious experiences in behaviorally non-responsive patients. *Neuroimage,* 145**,** 304-313.

REISBERG, B., FERRIS, S. H., DE LEON, M. J. & CROOK, T. 1982. The Global Deterioration Scale for assessment of primary degenerative dementia. *Am J Psychiatry,* 139**,** 1136-9.

ROCCHI, L., DI SANTO, A., BROWN, K., IBANEZ, J., CASULA, E., RAWJI, V., DI LAZZARO, V., KOCH, G. & ROTHWELL, J. 2021. Disentangling EEG responses to TMS due to cortical and peripheral activations. *Brain Stimul,* 14**,** 4-18.

ROGASCH, N. C., SULLIVAN, C., THOMSON, R. H., ROSE, N. S., BAILEY, N. W., FITZGERALD, P. B., FARZAN, F. & HERNANDEZ-PAVON, J. C. 2017. Analysing concurrent transcranial magnetic stimulation and electroencephalographic data: A review and introduction to the open-source TESA software. *Neuroimage,* 147**,** 934-951.

WHO 2016. *International statistical classification of diseases and related health problems (10th ed).* World Health Organization.
